# Recombinant COL6 α2 as a Self-Organization Factor That Triggers Orderly Nerve Regeneration Without Guidance Cues

**DOI:** 10.3389/fncel.2021.816781

**Published:** 2021-12-23

**Authors:** Zhou Fang, Jian-Long Zou

**Affiliations:** ^1^Institute of Neuroscience and the Second Affiliated Hospital of Guangzhou Medical University, Key Laboratory of Neurogenetics and Channelopathies of Guangdong Province and the Ministry of Education of China, Guangzhou, China; ^2^Key Laboratory of Neurological Function and Health, School of Basic Medical Sciences, Guangzhou Medical University, Guangzhou, China

**Keywords:** peripheral nerve injury, nerve bundle formation, self-organization, COL6 α2, immunogenicity, myelination

## Abstract

Collagen VI (COL6) in the microenvironment was recently identified as an extracellular signal that bears the function of promoting orderly axon bundle formation. However, the large molecular weight of COL6 (≈2,000 kDa) limits its production and clinical application. It remains unclear whether the smaller subunit α chains of COL6 can exert axon bundling and ordering effects independently. Herein, based on a dorsal root ganglion (DRG) *ex vivo* model, the contributions of three main COL6 α chains on orderly nerve bundle formation were analyzed, and COL6 α2 showed the largest contribution weight. A recombinant COL6 α2 chain was produced and demonstrated to promote the formation of orderly axon bundles through the NCAM1-mediated pathway. The addition of COL6 α2 in conventional hydrogel triggered orderly nerve regeneration in a rat sciatic nerve defect model. Immunogenicity assessment showed weaker immunogenicity of COL6 α2 compared to that of the COL6 complex. These findings suggest that recombinant COL6 α2 is a promising material for orderly nerve regeneration.

## Introduction

Peripheral nerve injury is a common disease for which there exist a large number of nerve repair strategies, including end-to-end neurorrhaphy, nerve grafting, gene therapy, and electrical stimulation. These medical and engineering approaches have shown effectiveness in promoting peripheral nerves regeneration (Gordon and English, 2016; Eggers et al., 2020; Raza et al., 2020). However, the disorganization of regenerated nerve fibers is still an important factor that leads to a mismatch between nerve fibers and target organs and restricts functional recovery from peripheral nerve injury (Robinson and Madison, 2004; English, 2005; de Ruiter et al., 2014).

The introduction of extrinsic guidance signals in nerve grafts is an effective strategy for orderly axon regeneration (Krick et al., 2011; Liu et al., 2018). However, these approaches require the anisotropic distribution of guidance signals, such as gradient-distributed biochemical cues or well-aligned physical cues, within small nerve grafts (Handarmin et al., 2011; Oh et al., 2018; Hsu et al., 2019). Hence, their axon-ordering effects are severely limited by the development of manufacturing techniques.

The concept of axonal self-organization without guidance cues may overcome this limitation. A previous study reported a unique phenomenon whereby dorsal root ganglion (DRG) axons tended to gather into straight bundles in the peripheral nerve extracellular matrix (ECM)-derived homogeneous hydrogel environment (Zou et al., 2018a). Collagen VI (COL6), an ECM protein abundant in various tissues, was subsequently identified as a microenvironmental cue that induced the formation of parallel nerve bundles through the neural cell adhesion molecule 1 (NCAM1)-mediated pathway (Sun et al., 2022).

In peripheral nerves, the COL6 is mainly secreted by Schwann cells, which in turn regulates the structure and function of Schwann cell. Lack of COL6 in the peripheral nervous system leads to increased myelin thickness and impaired motor coordination in mice (Cescon et al., 2015). Among the 28 types of identified collagens, COL6 is composed of COL6 α1, COL6 α2, and COL6 α3 chains, which undergo a unique process of supramolecular assembly leading to the formation of extremely large COL6 tetramers (molecular mass, ≈2,000 kDa) (Cescon et al., 2015). Due to the complicated assembly process and large molecular weight of COL6, it is difficult to prepare the whole COL6 protein using the recombinant protein technique. At the same time, the high molecular weight of COL6 also increases its immunogenicity (Lynn et al., 2004; Boeer et al., 2014). In general, whole proteins are not suitable drug candidates for several reasons, including susceptibility to proteolytic degeneration, antigenicity, and high associated costs. Therefore, some protein subunits or peptides are typically produced to achieve functional specificity, low immunogenicity, and to make drug candidates less costly (Ullrich et al., 1983; Lau and Dunn, 2018).

Encouraged by the results of these studies, here, we present a comparative study on the function and immunogenicity of recombinant COL6 α chains, which aimed to determine the independent effect of COL6 subunit chains on axonal self-organization with potential application in peripheral nerve injury.

## Materials and Methods

### Animals

Three to five day old Sprague–Dawley rats (with randomized sex; weights, 10–12 g) and 2-month-old Sprague–Dawley rats (female, ≈220 g) were purchased from the Animal Center Laboratory of Sun Yat-sen University (license no. SCXK (Yue) 2016-0029). All rats were housed under specific pathogen-free conditions. All animal experiments in this study were carried out per the ethical standards of the Animal Ethics Committee of Guangzhou Medical University (approval no. GY2019048) on April 30, 2019.

### Reagents

Human native COL6 protein (cat# 354261) and Matrigel (cat# 356230, growth factor reduced) were purchased from Corning Inc. (NY, USA). Pentobarbital (cat# P3761), Triton X-100 (cat# T8787), anti-neurofilament 200 (NF200) antibody (mouse monoclonal, cat# SAB4200705), anti-COL6 α3 antibody (rabbit polyclonal, cat# HPA010080), radioimmunoprecipitation assay (RIPA) buffer (cat# R0278) were purchased from Sigma–Aldrich (San Francisco, CA, USA). B27 (cat# 17504044) and L-glutamine (cat# 25030081) were purchased from Gibco (Grand Island, NY, USA). β-nerve growth factor (cat# 450-01) was purchased from Peprotech (Rocky Hill, NJ, USA). Anti-COL6 α1 antibody (rabbit monoclonal, cat# ab182744), anti-NCAM1 antibody (rabbit monoclonal, cat# ab220360), and rat IgM SimpleStep enzyme-linked immunosorbent assay (ELISA®) Kit (cat# ab215085) were purchased from Abcam (Cambridge, MA, USA). Anti-COL6 α2 antibody (mouse monoclonal, cat# sc-374566) was purchased from Santa Cruz Biotechnology, Inc. (Dallas, TX, USA). Anti-glyceraldehyde 3-phosphate dehydrogenase (anti-GAPDH) antibody (rabbit monoclonal, cat# AF1186), Cy3-conjugated goat anti-rabbit IgG (cat# A0516), Alexa Fluor 488-conjugated goat anti-mouse IgG (cat# A0428), bicinchoninic acid (BCA) protein assay kits (cat# P0009), horseradish peroxidase (HRP)-conjugated goat anti-mouse IgG (cat# A0216), HRP-conjugated goat anti-rabbit IgG (cat# A0208), anti-β-actin antibody (mouse monoclonal, cat# AA128), and fast silver stain kit (cat# P0017S) were purchased from Beyotime Biotechnology (Shanghai, China). Normal goat serum (cat# 31873) was purchased from Invitrogen (Carlsbad, CA, USA). Mounting medium containing 4′, 6-diamidino-2-phenylindole (DAPI) (cat# S36973), MAX Efficiency^TM^ DH5α cells (cat# 18258012), and a protein ladder (cat# 26625) were purchased from Thermo Fisher Scientific (Waltham, MA, USA). Myc-DDK-tagged human COL6 α2 open reading frame (ORF) clone (cat# RC209476) was purchased from OriGene Technologies Inc. (Rockville, MD, USA). HEK293 cells were purchased from the American Type Culture Collection (Manassas, VA, USA). Anti-Myc tag affinity gel (cat# IP0097) was purchased from Dia-An Biotechnology Co., Ltd. (Wuhan, Hubei, China). Protease/phosphatase inhibitor cocktail (cat# 5872S) was purchased from Cell Signaling Technology (Danvers, MA, USA). Sodium dodecyl sulfate-polyacrylamide gel electrophoresis (SDS-PAGE; cat# 4568123) was purchased from Bio-Rad (Hercules, CA, USA). Isoflurane (cat# R510-22-8) and a small animal anesthesia machine (R540) were purchased from RWD Life Science Co. Ltd. (Shenzhen, China). A silicone catheter (with diameter 1.2 mm) was purchased from Merck KGaA (Darmstadt, Germany).

### Coating of the Cell Culture Plates

In coating the culture plates with COL6, human native COL6 was dissolved in cold saline to obtain a 20 μg/ml COL6 solution before coating. The COL6 solution was added to the culture dishes at 2 μg/cm^2^. The culture dishes were placed at 37°C for 2 h and washed once with the culture medium before DRG implantation. Using the same procedure described above, the plates used in the COL6 α2 treatment group were precoated with a solution consisting of 1 mg/ml Matrigel and 10 μg/ml COL6 α2 in cold saline, and the plates used in the vehicle group were precoated with a solution containing 1 mg/ml Matrigel in cold saline.

### DRG *ex vivo* Preparation

Neonatal rats were deeply anesthetized via intraperitoneal injection with pentobarbital (200 mg/kg) before dissection of the DRG tissue blocks. The DRGs were then seeded onto protein-precoated dishes and immersed in Neurobasal-A medium containing 2% B27, 0.5 mM L-glutamine, and 50 ng/ml β-nerve growth factor. The dishes were placed in an incubator with a temperature of 37°C with the medium changed every 2 days.

### Antibody Blocking Assay

To block the function of COL6 subunit chains, the DRGs seeded on the COL6-precoated surface were incubated with function-blocking antibodies to COL6 α1, α2, and α3, respectively, at 37°C for 48 h. In other experiments, the DRGs growing on the Matrigel and COL6 α2-precoated surface were treated with an NCAM1 antibody for 48 h; then, the DRGs were fixed and examined by microscopy. An antibody against GAPDH was used as a control. All antibodies were applied at the same concentration (1 μg/ml) in this assay.

### Immunofluorescence Staining

After a period of incubation, the DRG tissue blocks were fixed with 4% paraformaldehyde for 2 h and thoroughly rinsed with phosphate-buffered saline for 30 min. The membrane was permeabilized by incubation in 0.3% Triton X-100 for 10 min, and non-specific antigens were blocked by incubation for 30 min with 10% normal goat serum. The specimens were then incubated with anti-NF200 antibody (1:200) or anti-NCAM1 antibody (1:300) at 37°C for 3 h, followed by incubation with Cy3-conjugated secondary antibody (1:500) or Alexa Fluor 488-conjugated secondary antibody (1:500) at 37°C for 1 h. The nuclei were visualized using a mounting medium containing DAPI.

### Measurement of Axon Diameter

In a subset of DRG *ex vivo* preparations, DRG tissue blocks with regenerated axons were photographed using a microscope at a magnification of 400 ×. The region of interest (ROI) was defined as a square with a length of 200 μm and randomly set at a distance of 500 μm from the DRG tissue block. Within the ROI, the diameter of each axon or bundle was measured, and six ROIs were recorded in each culture well; five biological replicates were performed for each group and the average diameter of the axon bundle in each group was calculated.

### Evaluation of Axon Directional Stability

Based on the micrographs of DRG *ex vivo* preparations, a 20 μm wide stripe was masked along the initial direction of the axon or axon bundle. The distance at which the axon or bundle first crossed the boundary of the stripe was measured, and a longer distance reflected better directional stability of the regenerating axon. The data for 20 axons were randomly recorded in each culture well and five biological replicates were performed for each group.

### Recombinant Protein Expression and Purification

The plasmid of the Myc-DDK-tagged human COL6 α2 ORF clone was purchased from OriGene. The product manual (https://cdn.origene.com/assets/documents/trueorf/trueorfapplicationguide.pdf) describes the protein expression method in detail. Briefly, the plasmid was first amplified in DH5α cells and then used to infect HEK293 cells to produce the Myc-DDK-tagged COL6 α2 chain. Seventy-two hours after transduction, the cells were harvested and lysed in a lysis buffer containing 150 mM sodium chloride, 50 mM tris(hydroxymethyl)aminomethane hydrochloride, 1% Triton X-100, and 5 mM ethylenediaminetetraacetic acid. The Myc-DDK-tagged COL6 α2 was separated via incubation with the anti-Myc tag affinity gel at room temperature for 2 h and then eluted using 0.15 M glycine hydrochloride buffer (pH 3.0). The purity of COL6 α2 in the eluent was detected by silver staining, and the pH of the COL6 α2 eluent was neutralized by dialysis against distilled water at 4°C overnight. After freeze-drying, the purified COL6 α2 was collected and stored as a lyophilized powder.

### Silver Staining of SDS-PAGE Gel

The following steps were performed according to the instructions of the fast silver stain kit. Briefly, after electrophoresis, the gel was rinsed with distilled water. We then fixed the gel in a fixation solution (50% v/v ethanol, 10% v/v acetic acid, 40% v/v ultrapure water) for 20 min and washed the gel in 30% ethanol while shaking for 10 min. The gel was rinsed with ultrapure water for 10 min. The gel was incubated in sensitizer solution for 1 min, followed by rinsing with two changes of ultrapure water for 1 min each, after which we incubated the gel in staining solution for 10 min. The gel was washed with two changes of ultrapure water while shaking for 20 s each. The developer solution was immediately added and incubated at room temperature while shaking until protein bands appeared (approximately 2–3 min). When the desired band intensity was reached, the developer working solution was replaced with a stop solution.

### Western Blot Analysis of NCAM1

In brief, DRGs were lysed in RIPA buffer containing 1% protease/phosphatase inhibitor cocktail. The protein concentration of the lysate was determined using the BCA assay. Protein samples (20 μg per well) were loaded onto an SDS-PAGE gel. After electrophoresis and transfer, the membrane was incubated with anti-NCAM1 antibody (1:1,000) and anti-β-actin antibody (1:1,000) overnight at 4°C, followed by incubation with HRP-conjugated goat anti-rabbit IgG (1:1,000), and HRP-conjugated goat anti-mouse IgG (1:1,000) for 1 h at room temperature. The protein bands were detected via electrochemiluminescence using a JS-M6 Chemiluminescence Imaging System (Peiqing Science and Technology Co. Ltd., Shanghai, China). The Image Lab software (version 6.0; Bio-Rad) was used to quantify the protein expression levels. The proteins of interest were normalized to β-actin. A protein ladder was used to determine the molecular weights.

### Sciatic Nerve Defect and Repair Model

Adult rats were anesthetized with isoflurane (2%, 0.6 L/min) using a small animal anesthesia machine (R540; RWD Life Science Co. Ltd.). The sciatic nerve on the right side was exposed and transected twice to create an 8 mm defect in the nerve trunk. The stumps were then bridged with a silicone catheter (with diameter 1.2 mm) filled with different types of hydrogels. In the COL6 α2 treatment group, the hydrogel was composed of 10 mg/ml Matrigel and 20 μg/ml COL6 α2, whereas in the vehicle group, the hydrogel was composed of 10 mg/ml Matrigel and an equal volume of saline. After surgery, the rats were housed under specific pathogen-free conditions at a temperature of 22 ± 1°C.

### Morphological Analysis of Sciatic Nerve Regeneration

Eight weeks postoperatively, the rats were euthanized with an overdose of isoflurane. Sciatic nerves were dissected from the animals and fixed in 4% paraformaldehyde for 48 h. Gradient sucrose dehydration and cryo-sectioning were performed to prepare longitudinal slices of the sciatic nerve at a thickness of 20 μm. Immunofluorescence staining of NF200 and NCAM1 was performed on these slices, as described above. Based on the fluorescence images, the density of axons along the transverse line was calculated, and the continuous length of axons was also measured and compared between different groups.

### Functional Experiments on the Regenerated Sciatic Nerves

The animals were anesthetized with isoflurane after 8 weeks of regeneration, and the catheter-bridged sciatic nerve and sham-operated sciatic nerve were carefully exposed. Using the BL-420 biological function experiment system (Techman Software Co. Ltd., Chengdu, China), a recording electrode was inserted into the gastrocnemius, and a stimulating electrode was placed sequentially at the proximal and distal parts of the injured nerve trunk, across the catheter. The electrical impulses were set at 8 V and 30 Hz, and traces of compound muscle action potential (CMAP) were recorded in different groups. The amplitude recovery was calculated by dividing the maximum amplitude in the experimental group by the maximum amplitude in the sham group. The motor nerve conduction velocity was calculated by dividing the difference between the latency of the proximal stimulus and the latency of the distal stimulus by the distance between the proximal and distal stimuli.

### Transmission Electron Micrography

Sciatic nerve tissues were fixed with 0.2 M phosphate buffer containing 2% paraformaldehyde and 2% glutaraldehyde, embedded in EPON-812. Transverse sections of sciatic nerve tissues were prepared via ultramicrotomy. After lead-uranium staining, the sections were mounted and photographed using a transmission electron microscope (Philips XL30 FEG; Philips, Eindhoven, The Netherlands). The density of myelinated axons was calculated, and the degree of myelination was evaluated using the axon-to-fiber diameter ratio, with a smaller ratio representing a higher degree of myelination.

### Immunogenicity Assessment of COL6 α2

To characterize the effect of human COL6 α2 on the production of inflammation-related cytokines in rats, the animals were immunized with COL6 α2 via subcutaneous injection of COL6 α2 (100 μg/ml, 0.5 ml) at the dorsal sites. After immunization for 3, 7, and 14 days, the tails were incised to collect 0.5 ml blood in a 1.5 ml Eppendorf tube. After 3 h of coagulation at room temperature, the serum was collected by centrifugation at 800 × g for 5 min. The sera collected before immunization were defined as the 0 day group. The levels of plasma cytokines (IL-1α, IL-1β, IL-2, IL-4, IL-5, IL-6, IL-7, IL-10, IL-12, IL-13, IL-17A, IL-18, GM-CSF, GRO/KC, IFN-γ, MCP-1, MIP-1α, MIP-3α, RANTES, TNF-α, VEGF, G-CSF, and M-CSF) were measured using the Rat Cytokine 23-Plex Assays panel and the Bio-Plex MAGPIX System (Bio-Rad) according to the manufacturer's instructions. Serum samples from COL6 (100 μg/ml, 0.5 ml) immunized rats were used as controls for comparison.

The primary humoral immune response was determined using the Rat IgM SimpleStep ELISA Kit according to the manufacturer's instructions. In brief, rat serum was diluted (1:10,000) with sample diluent, 50 μl of each sample was added to the wells, followed by mixing with 50 μl of the antibody cocktail. After 1 h incubation at room temperature, each well was washed with Wash Buffer and repeated three times. we added 100 μl of 3,3′, 5,5′-tetramethylbenzidine development solution to each well and incubated for 10 min on a plate shaker set to 400 rpm, then 100 μl of stop solution was added. The optical density of each well was recorded at 450 nm using an Infinite M200 PRO microplate reader (TECAN, Männedorf, Zürich, Switzerland).

### Heatmap Plotting

Matrix data of cytokine expressions were analyzed via an online heatmap plotting software (https://international.biocloud.net/en/software/tools/detail/small/305). The data were normalized by column, and we take the logarithm of the data (log base 10) for heatmap output.

### Statistical Analysis

Continuous variables were compared between the two groups using an unpaired *t-*test if the data had a Gaussian distribution and had the same standard deviation, or a ratio paired *t*-test if the ratios of paired values were consistent. Differences among three or more groups were compared via one-way analysis of variance, followed by Tukey's multiple comparison tests. All statistical tests were performed and plots were generated using the GraphPad Prism version 8 (GraphPad Software, LLC, San Diego, CA, USA). Analysis items with a two-tailed *p*-value < 0.05 were considered statistically significant.

## Results

### Contributions of COL6 Subunits in the COL6-Induced Orderly Axon Bundle Formation

**Figure 1:**
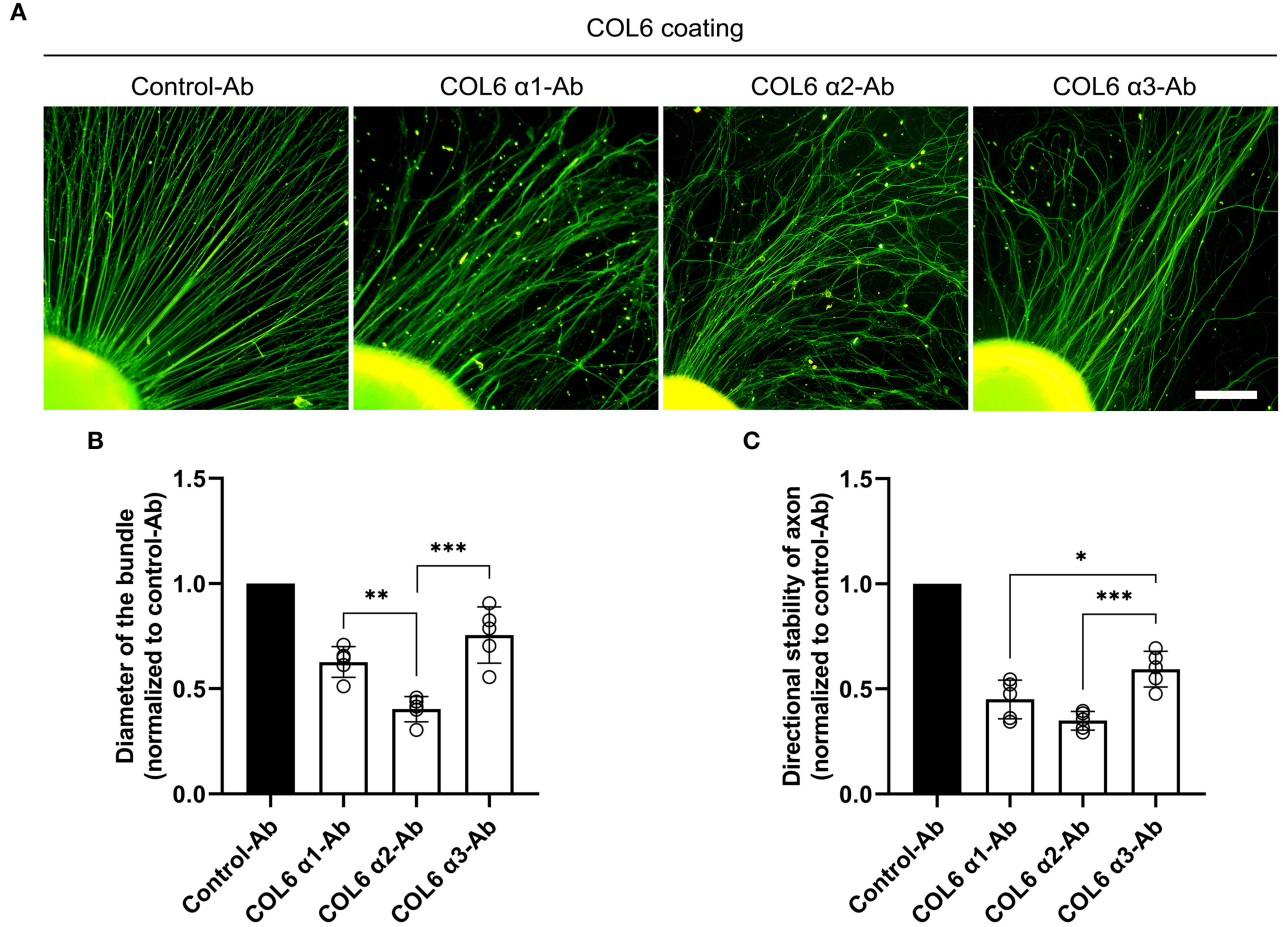
Function-blocking of different COL6 α chains hinders nerve bundle formation in various degrees. DRGs cultured on the COL6-coated surface were subjected to an antibody-blocking assay. **(A)** DRGs treated with control-antibody (an antibody against GAPHD) exhibited radially aligned axon bundles. The antibodies to different COL6 α chains gave rise to disorganized axons in varying degrees. **(B)** The relative values of axon bundle diameters are compared among different experimental groups (*n* = 5 independent biological experiments, **p = 0.0074, ***p = 0.0002, one-way ANOVA followed by Tukey's multiple comparison tests). **(C)** Histogram showing the relative values of directional stability of axons in different groups (*n* = 5 independent biological experiments, *p = 0.0287, ***p = 0.0008, one-way ANOVA followed by Tukey's multiple comparisons tests). Scale bar = 200 μm.

Dorsal root ganglions seeded on the COL6-coated substrate gradually exhibited well-aligned axon bundles after 48 h of incubation (Supplementary Video 1). The effects of function-blocking antibodies against different COL6 α chains on nerve bundle formation were examined. The control antibody against GAPDH did not hinder the formation of orderly nerve bundles. In contrast, the formation of orderly axon bundles was specifically inhibited by antibodies against COL6 α1 and COL6 α2; the antibody to COL6 α3 also exhibited a relatively weak inhibitory effect on the fasciculation and alignment of axons (Figure 1A). Morphological analysis showed that compared with the control antibody to GAPDH, the function-blocking antibodies to COL6 α1, α2, and α3 reduced the diameter of the bundle by 38, 60, and 22%, respectively (Figure 1B), and the directional stability of axons were decreased by 55, 65, and 41% in the COL6 α1, α2, and α3 blocking groups, respectively (Figure 1C). These results suggested that there were differences in the contribution of COL6 subunit chains to axon bundle formation, with COL6 α2 having the largest contribution weight.

### Expression of Recombinant Proteins of Human COL6 α2 Chain

**Figure 2:**
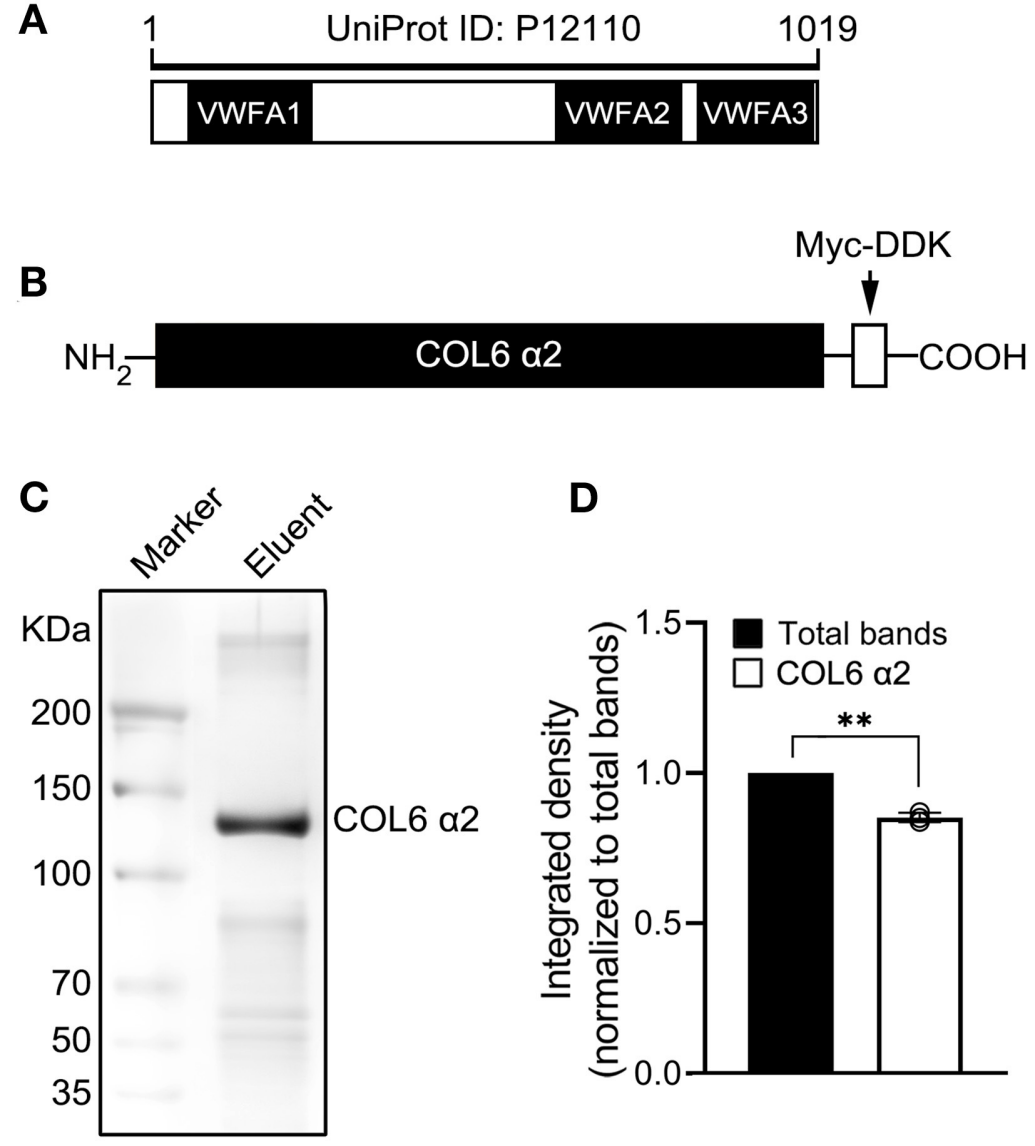
Schematic diagram of the COL6 α2 chain domains and the recombinant COL6 α2 protein used in this study. **(A)** The scale of amino acid is shown on the top. The COL6 α2 full-length chain (UniProt ID: P12110) is mainly composed of VWFA1, VWFA2, and VWFA3 domains. **(B)** Schematic diagram of the recombinant COL6 α2 full-length chain. A Myc-DDK tag was added at the C-terminal of the COL6 α2 chain. **(C)** After purification, the purity of the Myc-DDK-tagged COL6 α2 was analyzed via SDS-PAGE and visualized by silver staining. **(D)** The statistical analysis based on optical density of the protein bands determined that the purity of the recombinant COL6 α2 was 85.24 ± 1.56% (*n* = 3 independent biological experiments, **p = 0.0043, ratio paired *t*-test).

To investigate the independent functions of COL6 α2 in nerve bundle formation, a recombinant human COL6 α2 full-length chain was produced using a TrueORF clone among HEK293T cells. The amino acid sequence and major domains of the recombinant COL6 α2 protein are shown in Figure 2A. A Myc-DDK tag was added at the C-terminal of COL6 α2 to facilitate subsequent purification and identification of this protein (Figure 2B). The purity of this fusion protein was over 85% after purification with anti-Myc agarose under denaturing conditions, as determined via silver staining of SDS-PAGE gel (Figures 2C,D).

### Axon Clustering and Straightening Effects of Recombinant Human COL6 α2

**Figure 3:**
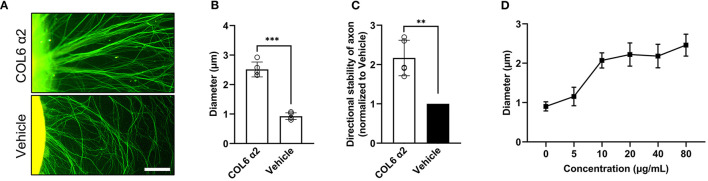
COL6 α2 treatment increases the diameter and directional stability of axon bundle in Matrigel. In the COL6 α2 group, DRGs were seeded on a surface precoated with normal saline solution containing Matrigel (1 mg/ml) and COL6 α2 (10 μg/ml), and in the vehicle group, the surface for DRG seeding was precoated with normal saline solution containing only Matrigel (1 mg/ml). (**A**) Representative immunofluorescence images of DRG axons (labeled with NF200, green, stained with Alexa Fluor 488) in different groups. **(B)** Histogram of average diameter showing increased axon bundle diameter in the COL6 α2, compared with that in the vehicle group (*n* = 5 independent biological experiments, ***p < 0.0001, unpaired *t-*test). **(C)** Histogram showing more than twice the directional stability of axons in the COL6 α2 group compared to the vehicle group (*n* = 5 independent biological experiments, **p = 0.0012, ratio paired *t-*test). (**D**) Different concentrations of COL6 α2 were tested for coating in the COL6 α2 group. The dose-dependent curve shows that the axon bundle diameter increased with increasing COL6 α2 concentrations (*n* = 5 independent biological experiments). Scale bar = 20 μm in **(A)**.

As described previously, a Matrigel-coated substrate was used to induce regeneration of dispersed and curved axons (Sun et al., 2022). The addition of COL6 α2 (10 μg/ml) to the solution of Matrigel (1 mg/ml) for coating led to the formation of orderly axon bundles, while the addition of a vehicle (an equal volume of saline) to Matrigel still resulted in disordered axons (Figure 3A).

Morphological analysis revealed approximately three times the average diameter of bundles in the COL6 α2 group (2.51 ± 0.25 μm) compared with that in the vehicle group (0.93 ± 0.11 μm, Figure 3B). The directional stability of axons in the COL6 α2 group was significantly better than that in the vehicle group (Figure 3C). The increase in axon bundle diameter with COL6 α2 occurred in a dose-dependent manner (Figure 3D).

These results demonstrated an independent role of the recombinant COL6 α2 chain in axonal self-organization.

### Involvement of NCAM1 in the COL6 α2-Induced Axonal Alignment and Fasciculation

NCAM1 has been demonstrated to mediate axon bundle formation (Sun et al., 2022). We speculated that COL6 α2-induced axonal self-organization may also require the participation of NCAM1.

**Figure 4:**
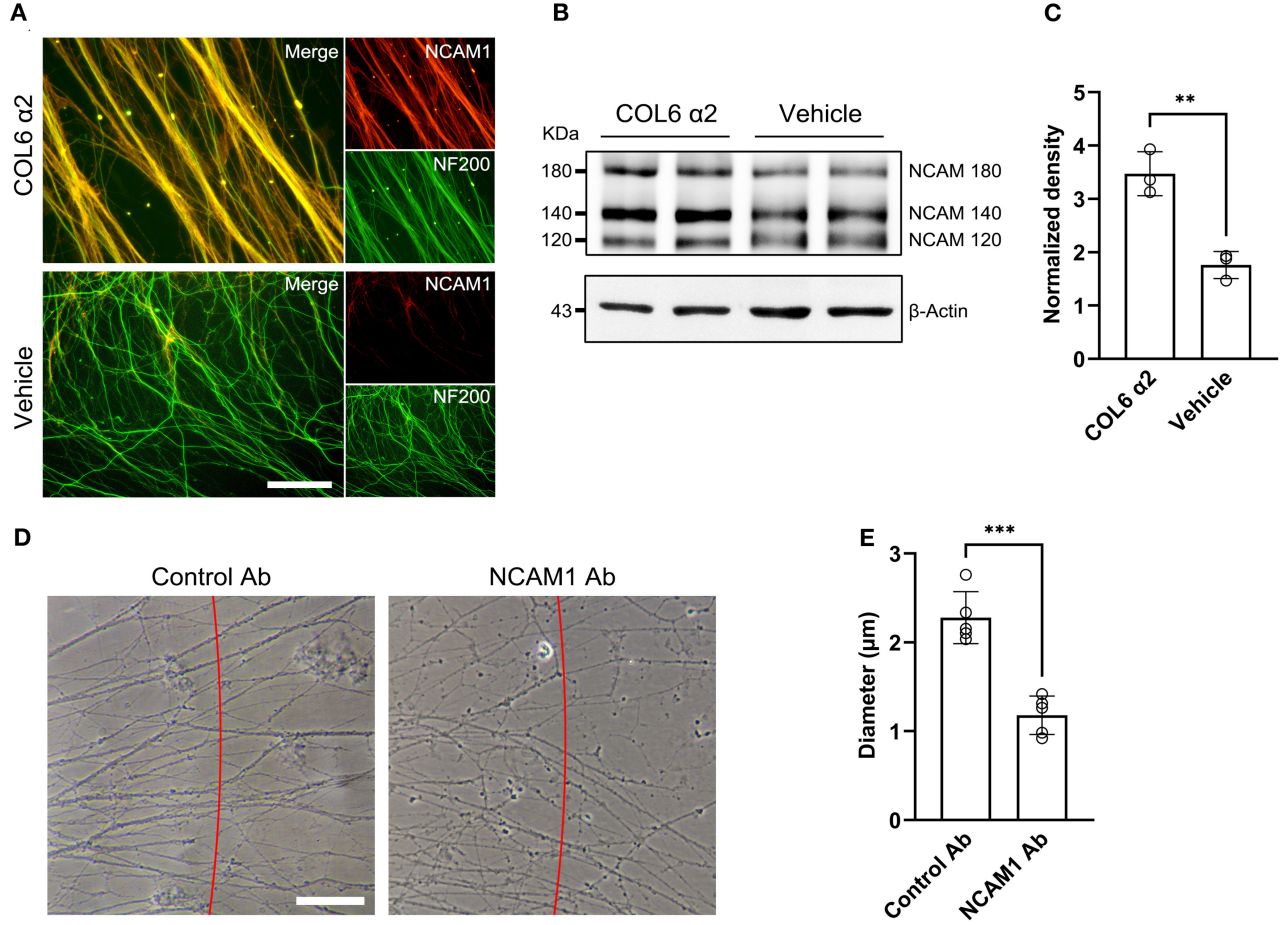
COL6 α2 promotes axonal clustering through the NCAM1-mediated pathway. **(A)** Representative immunofluorescence images showing the expression of NCAM1 (red) in axons (labeled with NF200, green). The fluorescence signal of NCAM1 was stronger in the COL6 α2 group than in the vehicle group. **(B)** Immunoblotting exhibited the NCAM1 (NCAM120, NCAM140, and NCAM180) bands together with the β-actin bands in the COL6 α2 and vehicle groups. **(C)** Histogram of the normalized density showing an upregulated NCAM1 expression in the COL6 α2 group, compared with the vehicle group (*n* = 3 independent experiments, **p = 0.0036, unpaired *t-*test; the expression of NCAM1 was normalized to β-actin). **(D)** Microscopy of axon bundles growing on the COL6 α2-coated substrate followed by control-antibody (GAPDH-Ab) or NCAM1-antibody blocking for 48 h. The red line indicates a 500 μm distance from the DRG tissue block. The diameter of each bundle across the red line was measured. **(E)** Histogram showing a significantly decreased axon bundle diameter with NCAM1-antibody treatment, compared with the control-antibody (*n* = 5 independent biological experiments, ***p < 0.0001, unpaired *t-*test). Scale bar = 20 μm in **(A)**.

Immunofluorescent staining of DRG *ex vivo* preparations showed higher axonal NCAM1 expression in the COL6 α2 group than in the vehicle group (Figure 4A). Consistent with the results of the immunofluorescence assay, western blot assays confirmed the upregulation of three NCAM1 isoforms (NCAM180, NCAM140, and NCAM120) in the COL6 α2 treatment group compared with the vehicle group (Figures 4B,C). Antibody blocking assays showed that compared with the control antibody to GAPDH, the function-blocking antibodies to NCAM1 significantly hindered the formation of axon bundles induced by COL6 α2 (Figures 4D,E).

### *In vivo* Functions of Recombinant COL6 α2 Chain

Next, the function of the recombinant COL6 α2 protein was tested in a rat sciatic nerve defect model. Silicone catheters filled with Matrigel (10 mg/ml) were used to bridge the nerve stumps. An additional COL6 α2 (20 μg/ml) was mixed with Matrigel in the COL6 α2 treatment group, and an equal volume of saline was mixed with Matrigel in the vehicle group.

**Figure 5:**
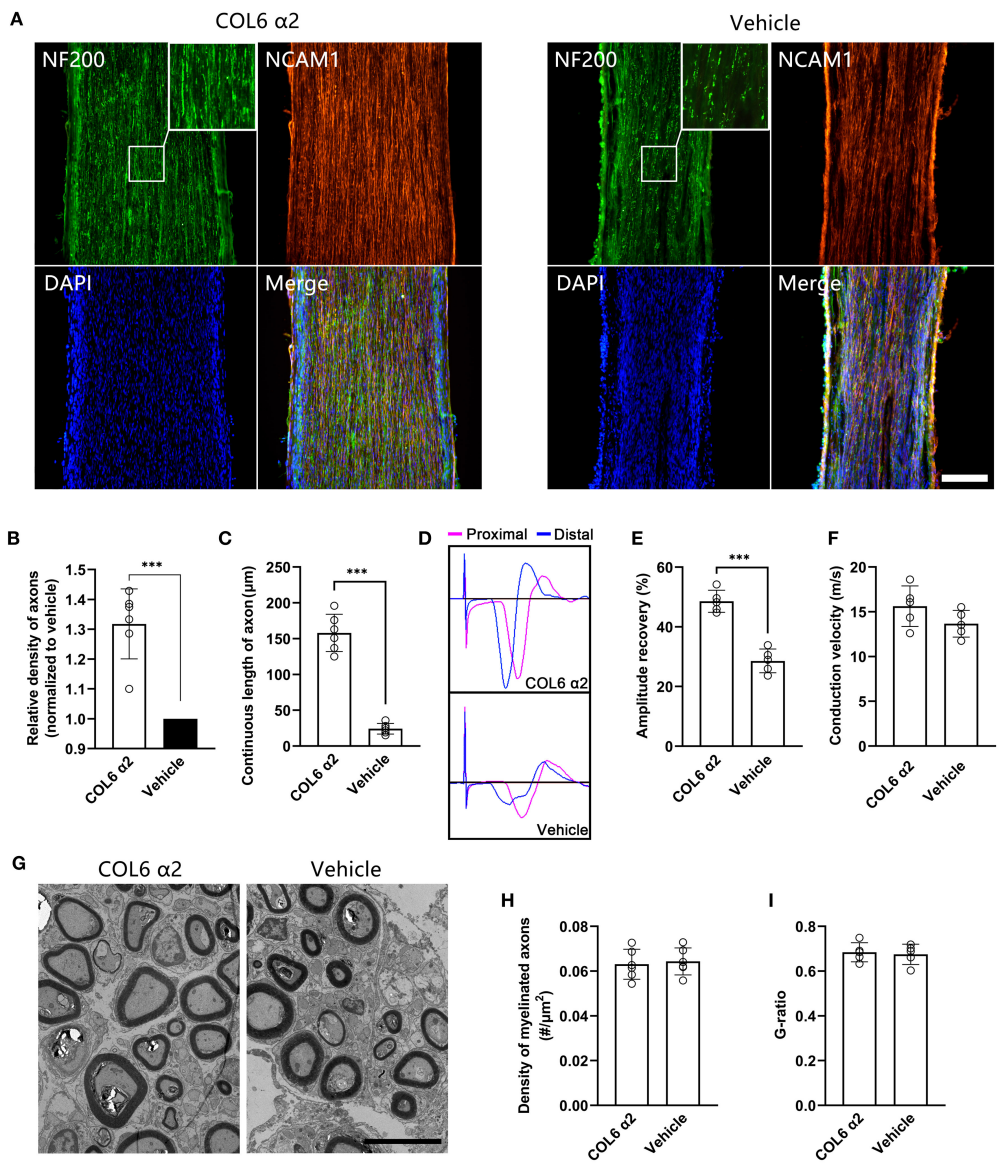
Addition of COL6 α2 in Matrigel improves the regeneration of sciatic nerve defect. Based on a rat sciatic nerve defect model, the nerve stumps were bridged with a catheter filled with 10 mg/ml Matrigel as the carrier. 20 μg/ml COL6 α2 and an equal volume of saline were added to the Matrigel in the COL6 α2 and vehicle groups, respectively. **(A)** After 8 weeks of repair, the longitudinal sections with immunofluorescence staining showed regenerated nerve tissues from the COL6 α2 and vehicle groups. **(B)** Histogram showing an increased density of axons in the COL6 α2 group compared to the vehicle group (*n* = 6 animals, ***p = 0.0009, ratio paired *t-*test, normalized to vehicle). **(C)** The continuous length of axons was measured and compared between the two groups (*n* = 6 animals, ***p < 0.0001, unpaired *t-*test). **(D)** Panels showing representative traces of CMAP in the COL6 α2 and vehicle groups (magenta trace, the stimulating electrode was placed at the proximal of the nerve trunk; blue trace, the stimulating electrode was placed distal to the nerve trunk). **(E)** Histogram showing the amplitude recovery of the injured sciatic nerve in the COL6 α2 and vehicle groups (*n* = 5, ***p < 0.0001, unpaired *t-*test). **(F)** Histogram showing the motor nerve conduction velocities of the COL6 α2 and vehicle groups; no statistical difference was detected between the two groups (*n* = 5, *p* = 0.1449, unpaired *t-*test). **(G)** Representative TEM images of sciatic nerve cross-sections showing myelinated axons after 8 weeks of regeneration. **(H)** Histogram showing the density of myelinated axons in the COL6 α2 and vehicle groups (*n* = 6 visual fields from three animals, *p* = 0.7378, unpaired *t-*test). **(I)** The degree of myelination was evaluated between groups using the axon-to-fiber diameter ratio (G-ratio; *n* = 5 visual fields, *p* = 0.7442, unpaired *t-*test). Scale bars = 200 μm in **(A)** and 5 μm in **(G)**.

After 8 weeks of repair, immunofluorescent staining showed that the NF200-positive axons were fasciculated and evenly distributed in the longitudinal sections from the COL6 α2 group. In contrast, axons were scattered and laterally distributed in the vehicle group (Figure 5A). The regenerated sciatic nerve tissues also exhibited higher levels of NCAM1 expression in the COL6 α2 group than in the vehicle group.

Morphological analysis based on the immunofluorescence images showed that COL6 α2 treatment resulted in more than 1.3 times the axon density observed with vehicle treatment (Figure 5B). The mean continuous length of axons was 158 ± 25.97 μm in the COL6 α2 group vs. 24.17 ± 7.36 μm in the vehicle group (Figure 5C), which represented increased stability of axon direction with COL6 α2 treatment.

Functionally, vehicle treatment led to a 26.19 ± 2.65% recovery in sciatic nerve CMAP following 8 weeks of regeneration, and the addition of 20 μg/ml COL6 α2 to Matrigel enhanced the recovery of CMAP by up to 49.55 ± 4.73% (Figures 5D,E). However, the motor nerve conduction velocities were not significantly changed by COL6 α2 administration (Figure 5F). Consistent with this, transmission electron microscopy revealed a comparable level of myelination in the regenerated nerve tissues between the COL6 α2 and vehicle groups (Figures 5G–I).

### Comparison of the Immunogenicity of COL6 α2 and COL6

**Figure 6:**
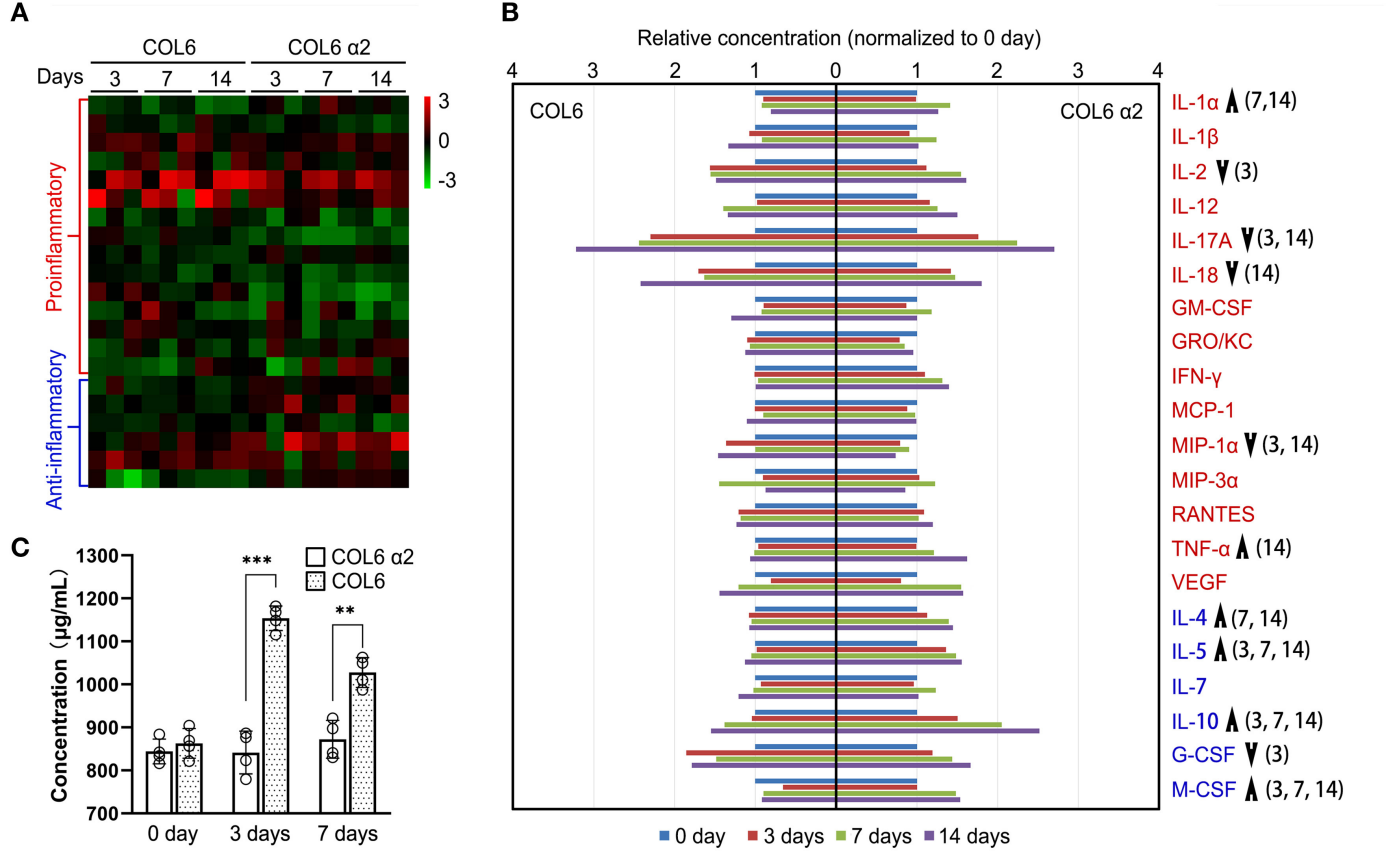
Immunogenicity assessment of human-sourced COL6 α2 and COL6 among rats. **(A)** Heatmap of cytokine expressions in COL6 and COL6 α2 immunized rat serum at 3, 7, and 14 days; the order of cytokines was consistent with that in **(B)**. **(B)** Relative expression levels of each pro-inflammatory cytokine (red font) and anti-inflammatory cytokine (blue font) were compared between COL6 and COL6 α2. Up and down arrows respectively indicate higher and lower levels of cytokine in COL6 α2, compared with COL6. The numbers in brackets indicate statistical differences between the groups on specific days [three independent biological experiments were performed in **(A)** and **(B)**]. **(C)** Concentration of serum IgM at various time points, detected via ELISA (*n* = 4 independent biological experiments, ***p < 0.0001, **p = 0.0015 unpaired *t*-test).

To assess the immunogenicity of human COL6 α2 and COL6 in rats, the release of 23 inflammatory cytokines in rat serum was examined using a Luminex system, following subcutaneous injection of COL6 α2 and COL6. A heat map showed that the overall expression levels of pro-inflammatory factors were relatively lower, and the levels of anti-inflammatory factors were relatively higher in the COL6 α2 group than in the COL6 group (Figure 6A). Among them, there were significant differences in four down-regulated (IL-2, IL-17A, IL-18, and MIP-1α) and two up-regulated (IL-1α and TNF-α) pro-inflammatory factors, and four up-regulated (IL-4, IL-5, IL-10, and M-CSF) and one down-regulated (G-CSF) anti-inflammatory factors (Figure 6B). It is noteworthy that among all pro-inflammatory factors detected, IL-17A showed the highest increase after 14 days of immunization, 3.22 ± 1.70 and 2.70 ± 1.26 times higher than those recorded before immunization in the COL6 and COL6 α2 groups, respectively. In terms of anti-inflammatory factors, IL-10 showed the highest increase (2.52 ± 0.75 times that obtained before immunization) after subcutaneous injection of COL6 α2 for 14 days. IL-6 and IL-13 were excluded from the comparison because their concentrations were lower than the detection range of the standard curve.

ELISA revealed a significantly higher level of IgM, the main serum immunoglobulin prominent in the early immune response, at 3 and 7 days following subcutaneous injection of COL6, while subcutaneous injection of COL6 α2 resulted in no significant change in IgM within 7 days (Figure 6C).

## Discussion

The data from this study revealed that COL6 α2, one of the three subunit chains of COL6, plays an important role in the induction of orderly axon bundle formation. A recombinant protein of the human COL6 α2 full-length chain recapitulated the axon clustering and ordering effects of COL6, in that compared with COL6 complex, the COL6 α2 chain triggered a weaker immune response.

In the dynamic sense, the organization of orderly axon bundles can be divided into hetero-organization and self-organization. Hetero-organization is defined as the process of constructing system patterns according to pattern information introduced from outside the system (Wu and Nan, 2019). For example, the process by which axons grow along the parallel topology provided by a multichannel nerve catheter (Mobasseri et al., 2015; Chang et al., 2018; Song et al., 2020), and the growth cone turns in response to the spatiotemporal distribution patterns of biochemical guidance cues (Tang et al., 2013; Zou et al., 2018b). The most attractive advantage of hetero-organization in nerve repair is the customizable nerve regeneration route. However, sophisticated extrinsic pattern information is usually vulnerable to complex microenvironments *in vivo*, such as the destruction of the material structure by enzymes, and the deposition of ECM on the surface of the material may destroy the pattern information originally carried in the nerve grafts.

Self-organization, on the contrary, refers to a process in which the system spontaneously forms an internal orderly structure through interactions among its components rather than through external intervention or instruction (Haken and Portugali, 2017). Examples of this process include the generation of patterns on animal furs, fish skins, and butterfly wings (Liu et al., 2006; Werner et al., 2010; Konow et al., 2021); the development of fertilized eggs and embryos (Niu et al., 2019; Xiang et al., 2020); and the orderly axon bundle formation in the homogeneous, COL6- or COL6 α2-containing microenvironment without any guidance cues. This concept greatly simplifies the strategy for ordering nerve regeneration; however, it also has some limitations in precisely guiding the direction of axonal regeneration.

According to synergetics, the main component of self-organization theory, the synergy of elements inside the system comprises the basis of self-organization (Corning, 1995; Kröger, 2015). A previous study demonstrated that extracellular COL6 can directly bind to the fibronectin type 3 domain of NCAM1, resulting in the accumulation of NCAM1 molecules on axolemma, promoting axon bundle formation through their homophilic (NCAM–NCAM) binding activity (Sun et al., 2022). Therefore, the synergy between COL6 and NCAM1 is regarded as the key to the self-organization of the nerve bundle structure. The results of this study showed that the COL6 α2 chain uses the same NCAM1-mediated mechanism pathway as COL6.

In addition to the interaction of COL6 α1, α2, and α3 chains to form COL6 tetramers, the assembly of protein complex is a ubiquitous phenomenon in organisms. The united proteins can act in coordination to generate functions of which the individual proteins are incapable (Berg et al., 2002). On the other hand, some protein subunits such as β-NGF have been demonstrated to exert biological activities ascribed to the NGF complex in the development and preservation of the sensory and sympathetic nervous systems (Castellanos et al., 2003; Perrard and Durand, 2009). The PPFLMLLKGSTR motif within the laminin-5 α3 chain inherits the cell adhesion function of laminin-5 (Kim et al., 2005). These findings are in line with the results of the present study as they showed that the COL6 α2 chain had an independent function in orderly axon bundle formation.

To improve the efficiency of protein purification, we used a denaturing buffer during the purification of recombinant human COL6 α2 chains. Even after denaturation, COL6 α2 still maintained the ability to trigger axonal self-organization, which demonstrates a strong resistance of the recombinant COL6 α2 chain to environmental interference. In addition to nerve bundle formation, the number of axons in the regenerated nerve tissue was also increased by COL6 α2 treatment, indicating that COL6 α2 may also inherit other neuroprotective and immunomodulatory effects from COL6 (Urciuolo et al., 2013; Chen et al., 2015; Cescon et al., 2016).

In the peripheral nervous system, COL6 exhibits a dual myelination activity in a dose-dependent manner, the lack of COL6 in *Col*6*a*1^−/−^ mice causes hypermyelination (Chen et al., 2014), while a high concentration of COL6 in the microenvironment leads to the detachment of Schwann cells and axons (Sun et al., 2022). It is reasonable to consider that the addition of COL6 α2, a functional subunit of COL6, in the microenvironment may affect axonal myelination, and data from transmission electron microscopy and motor nerve conduction velocity assessment showed that the addition of 20 μg/ml COL6 α2 to Matrigel had no adverse effects on axonal myelination.

The present study detected lower levels of IgM secretion with COL6 α2 immunization compared to COL6 immunization, which points to a weaker humoral immune response to COL6 α2. Following subcutaneous injection of COL6 and COL6 α2, the pro-inflammatory factor IL-17A showed the most significant change among all the 21 inflammatory factors detected, suggesting that IL-17A can be used as an important index in evaluating the immune response induced by COL6 and COL6 α2. On the other hand, the increased expressions of several anti-inflammatory factors (particularly, IL-10) in the COL6 α2 treated group, indicated an advanced anti-inflammatory effect of COL6 α2 in comparison with the COL6 complex.

In terms of methodology, gene knockout strategies are widely used to study loss-of-function phenotypes (Zare et al., 2018; Freund et al., 2020; Paul et al., 2021). However, this approach is not suitable for determining the functional differences among COL6 subunit chains, because the COL6 α1, α2, and α3 chains must be assembled into COL6 tetramers in the cytoplasm before they can be secreted into the ECM. Knockout of the gene encoding any one of the COL6 α chains will result in the simultaneous disappearance of all three COL6 α chains in the ECM (Bonaldo et al., 1998; Irwin et al., 2003). Hence, an antibody blocking assay was preferred in the present study. In the section on immunogenicity assessment, considering that the inflammatory responses caused by the surgery and carrier material (Matrigel) may conceal the differences in immunogenicity between COL6 and COL6 α2, we performed subcutaneous injections to eliminate these interfering factors.

This study exhibits two distinct dynamics of axon clustering and straightening during axon self-organization, the homophilic binding of NCAM1 could explain the dynamic of axon clustering, but the detailed molecular mechanisms underlying axonal straightening remain to be further studied. The recombinant human COL6 α2 shows relatively low immunogenicity in rat, but we cannot completely rule out its potential allogeneic immunogenicity in human. Considering that COL6 is mainly secreted by Schwann cells and has regulatory effects on myelination, it may be a better solution to modulate the production of endogenous COL6 in the early stage of peripheral nerve injury through genetic engineering.

Collectively, the present study demonstrated the role of COL6 α2 in axonal self-organization, which leads to the formation of orderly axon bundles and promotes peripheral nerve regeneration. Based on the data presented, the recombinant COL6 α2 showed better feasibility and lower immunogenicity than the COL6 complex in practical applications, which indicates a promising prospect for clinical transformation.

## Data Availability Statement

The datasets generated or analyzed for this study can be found in the Google Drive, https://drive.google.com/drive/folders/1iHJS02jkUxQQhwza_PCW8-B3KmHMl6Be?usp=sharing.

## Ethics Statement

The animal study was reviewed and approved by Animal Ethics Committee of Guangzhou Medical University (approval no. GY2019048).

## Author Contributions

ZF and J-LZ designed the experiments and wrote the manuscript. ZF conducted the experiments and analyzed the data. All authors contributed to the article and approved the submitted version.

## Funding

This study was supported by grants from National Natural Science Foundation of China, No. 31800892 (to J-LZ).

## Conflict of Interest

The authors declare that the research was conducted in the absence of any commercial or financial relationships that could be construed as a potential conflict of interest.

## Publisher's Note

All claims expressed in this article are solely those of the authors and do not necessarily represent those of their affiliated organizations, or those of the publisher, the editors and the reviewers. Any product that may be evaluated in this article, or claim that may be made by its manufacturer, is not guaranteed or endorsed by the publisher.

## Supplementary Material

Supplementary Video 1 Axonal self-organization on the COL6-coated substrate.
